# Supplementary material for: Double-Lumen Endotracheal Tube—Predicting Insertion Depth and Tube Size Based on Patient’s Chest X-ray Image Data and 4 Other Body Parameters
Source: Diagnostics (Basel). 2022 Dec 14;12(12):3162. doi: 10.3390/diagnostics12123162 (PMC9777797; doi:10.3390/diagnostics12123162)
Supplement: Supplementary file 1 [file diagnostics-12-03162-s001.zip › diagnostics-2087319-supplementary.pdf]

**Table S1.** Without outlier rejection, we again compared the results of two models: linear regression and SVM, with full parameter sets or with leave-one-out. Note poorer performance compared with **Table 7**.

| Depth | Linear Regression | Cubic SVM |
|---|---|---|
| **4 Parameters** | 0.81 | 0.84 |
| **3 Parameters (no Sex)** | 0.74 | 0.82 |
| **3 Parameters (no Height)** | 0.74 | 0.76 |
| **3 Parameters (no TD-Trachea)** | 0.82 | 0.73 |
| **3 Parameters (no Weight)** | 0.81 | 0.75 |
| **Size** | **Linear Regression** | **Cubic SVM** |
| **5 Parameters** | 0.68 | 0.80 |
| **4 Parameters (no Sex)** | 0.63 | 0.70 |
| **4 Parameters (no Height)** | 0.68 | 0.69 |
| **4 Parameters (no TD-Trachea)** | 0.68 | 0.73 |
| **4 Parameters (no Chest circumference)** | 0.68 | 0.76 |
| **4 Parameters (no Weight)** | 0.67 | 0.77 |

**Table S2.** Without outlier rejection, results of two models (using linear regression and SVM, with full parameter sets or with leave-one-out) based alternatively on the top 5 parameters with r >0.65 show minimal improvements in performance.

| Depth | Linear Regression | Cubic SVM |
|---|---|---|
| **5 Parameters** | 0.80 | 0.86 |
| **4 Parameters (no Sex)** | 0.77 | 0.89 |
| **4 Parameters (no Height)** | 0.73 | 0.78 |
| **4 Parameters (no TD-Trachea)** | 0.81 | 0.82 |
| **4 Parameters (no TD-Cricoid)** | 0.81 | 0.86 |
| **4 Parameters (no APD-Cricoid)** | 0.81 | 0.83 |
| **Size** | **Linear Regression** | **Cubic SVM** |
| **5 Parameters** | 0.66 | 0.83 |
| **4 Parameters (no Sex)** | 0.63 | 0.74 |
| **4 Parameters (no Height)** | 0.65 | 0.72 |
| **4 Parameters (no TD-Trachea)** | 0.66 | 0.67 |
| **4 Parameters (no TD-Cricoid)** | 0.65 | 0.69 |
| **4 Parameters (no APD-Cricoid)** | 0.66 | 0.75 |

**Material S1**: Support Vector Machine (SVM)

In this study, a support vector machine (SVM) with a kernel cubic (polynomial) algorithm was used for classification prediction of intubation depth and tube size. Support Vector Machine (SVM) [19–25] is a kind of generalized linear classifier that performs binary classification on data according to supervised learning. Its decision boundary is the maximum-margin hyperplane solved for the learning samples. SVM uses the hinge loss function to calculate the empirical risk and adds a regularization term to the solution system to optimize the structural risk. It is a classifier with sparsity and robustness.

SVM can perform nonlinear classification through the kernel method, which is one of the common kernel learning methods

[4]. Its main idea is to transform the original input set to a high-dimensional feature space by using a kernel function, and then achieve optimum classification in this new feature space. Many kinds of implementation of SVM can be found on the Internet. The SVM technique of classification is useful when a dilemma of low memory space is faced. SVM finds a hyperplane in multidimensional space which divides the classes into best possible way. Here cubic SVM type classifier is employed where the kernel function of the classifier is cubic given as $k(x_i, x_j)$

$$k(x_i, x_j) = (x_i^T, x_j)^3 \tag{3}$$

Jingyu Xue [26] used support vector machines (SVM) to train on real data of 520 diabetic patients and potential diabetic patients aged 16 to 90 with an accuracy of 96.54%. Zayrit Soumaya [27] et al applied genetic algorithm and SVM to extract features from speech signals to detect some neurological diseases such as Alzheimer's disease, depression and Parkinson's disease. The best accuracy they got was 91.18%. In addition, the training parameters used in this study are: cubic kernel function, automatic kernel scale, box constraint level 1, one-vs-one multiclass method, standardize data, disabled hyperparameter options, all features used in the model.

**Material S2**: Robust Linear Regression

We use the intubation depth study as an example: each of the 4 body parameters (sex, height, TD-trachea and weight). We then implemented the linear regression analysis[18,28–30], as described below, to determine a linear equation that allowed the intubation depth best predicted by the 4 body parameters.

Linear simultaneous equations have the matrix representation as follows:

$$x = regression(b, A) \tag{1}$$

returns a vector b of coefficient estimates for a multiple linear regression of the responses in vector b on the predictors in matrix A. To compute coefficient estimates for a model with a constant term (intercept), a column of '1' is included in the matrix A. Where A is composed of the body parameters (e.g., sex, height, TD-trachea and weight etc.) and b is intubation depth

$$Ax = b$$

where A is a known matrix, b is a known row vector, and x is an unknown row vector. For simplicity, one assumes that the dimensions of A, x, and b are m*n, n*1, and m*1, respectively, where m represents the number of equations and n is the number of unknowns, which can be divided into three cases:

1: If m = n, it means that the number of equations and the number of unknowns are equal. In this case, there is typically a set of solutions x that satisfies Ax=b.

2: If m > n, it means that the number of equations is greater than the number of unknowns. In this case, there is typically no solution that satisfies Ax=b. But one can instead obtain the least squares solution (Least-Squares Solution) $\hat{x}$ which satisfies $\hat{x} = argmin_x |Ax - b|^2$.

3: If m < n, it means that the number of equations is smaller than the number of unknowns. In this case, there are typically an infinite number of solutions x that can satisfy Ax=b. We can seek a Basic Solution x so that x contains at least m-n Zero elements.

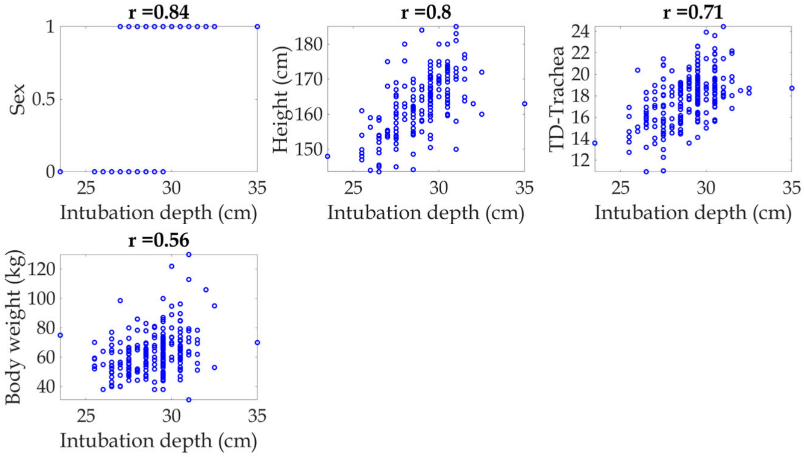


(**a**) before rejecting ‘depth’ outliers (n=231)

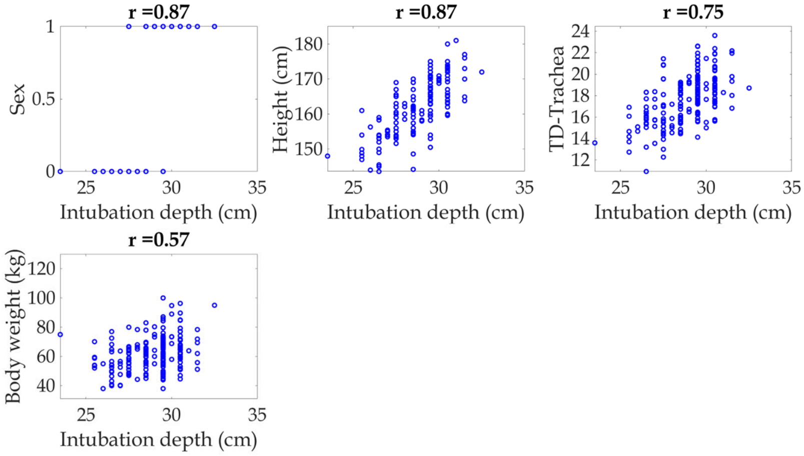


(**b**) after rejecting ‘depth’ outliers (n=193)

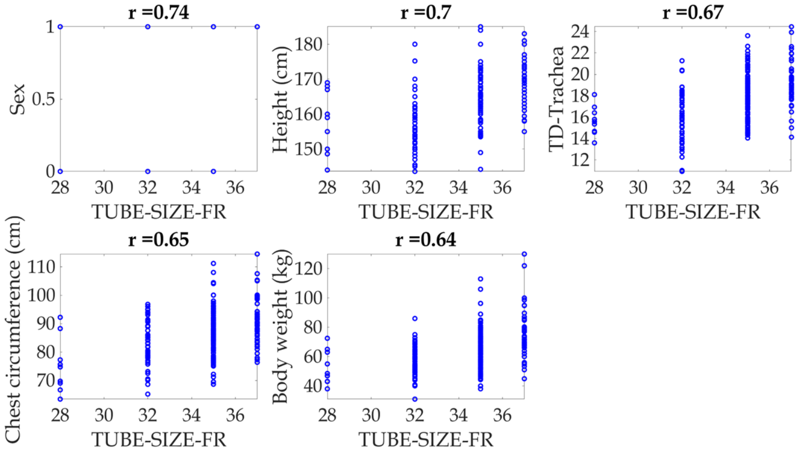


(**c**) before rejecting ‘size’ outliers (n=231)

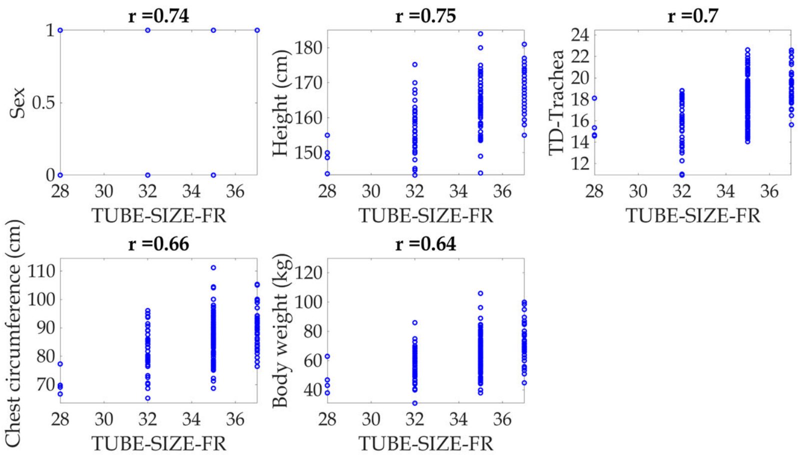


(**d**) after rejecting 'size' outliers (n=197)

**Figure S1.** Scatterplots of the 4 or 5 selected body parameters against 'depth' or 'size' values (**a**)(**c**) before and (**b**)(**d**) after rejection of outliers in separate accounts. Note some data symbols are overlapped.
